# Computational analysis of heat shock proteins and ferroptosis-associated lncRNAs to predict prognosis in acute myeloid leukemia patients

**DOI:** 10.3389/fgene.2023.1218276

**Published:** 2023-08-01

**Authors:** Fangfang Ge, Yulu Wang, Amit Sharma, Ulrich Jaehde, Markus Essler, Matthias Schmid, Ingo G. H. Schmidt-Wolf

**Affiliations:** ^1^ Department of Integrated Oncology, Center for Integrated Oncology (CIO), University Hospital Bonn, Bonn, Germany; ^2^ Department of Neurosurgery, University Hospital Bonn, Bonn, Germany; ^3^ Department of Clinical Pharmacy, Institute of Pharmacy, University of Bonn, Bonn, Germany; ^4^ Department of Nuclear Medicine, University Hospital Bonn, Bonn, Germany; ^5^ Institute for Medical Biometry, Informatics and Epidemiology, University Hospital Bonn, Bonn, Germany

**Keywords:** acute myeloid leukemia, heat shock proteins, ferroptosis, long noncoding RNAs, prognosis

## Abstract

Owing to their functional diversity in many cancers, long noncoding RNAs (lncRNAs) are receiving special attention. LncRNAs not only function as oncogenes or tumor suppressors by participating in various signaling pathways but also serve as predictive markers for various types of cancer, including acute myeloid leukemia (AML). Considering this, we investigated lncRNAs that may act as a mediator between two processes, i.e., heat shock proteins and ferroptosis, which appear to be closely related in tumorigenesis. Using a comprehensive bioinformatics approach, we identified four lncRNAs (AL138716.1, AC000120.1, AC004947.1, and LINC01547) with prognostic value in AML patients. Of interest, two of them (AC000120.1 and LINC01547) have already been reported to be AML-related, and AC004947.1 is considered to have oncogenic potential. In particular, the signature obtained showed a lower survival probability with high-risk patients, and *vice versa*. To our knowledge, this is the first predictive model of lncRNA that may correlate with the processes of heat shock proteins and ferroptosis in AML. Nevertheless, validation using patient samples is warranted.

## Introduction

It has been almost two decades since gene expression-based prognostic classification has been introduced in acute myeloid leukemia (AML). Undeniably, several approved approaches ranging from coding (Li et al., 2020a; Li et al., 2020b) to noncoding, including micro-RNA and long noncoding RNA (lncRNA) expression, have been successfully used to model patients’ stratifications in AML (Wallace and O’Connell, 2017; Gourvest et al., 2019; Singh et al., 2021). This, in turn, also raises the possibility of further integrating and understanding the unrelated molecular processes involved in different types of cancer, including AML.

In particular, lncRNAs have received considerable attention in recent years due to their involvement in developmental processes and various diseases, including AML. For instance, one study investigated the differential expression profiles of lncRNAs in AML patients by microarray and found that SNHG5 significantly regulates chemotherapy resistance in AML through the miR-32/DNAJB9 axis (Wang et al., 2020). Underexpression of LINC00649 has been reported to be an unfavorable prognostic marker in acute myeloid leukemia (Guo et al., 2020). Garzon et al. revealed that some deregulated lncRNAs were associated with recurrent mutations and clinical outcome in AML patients (Garzon et al., 2014). Serum LINC00899 was predicted to be a potential and useful noninvasive biomarker for the early clinical detection and prognosis of AML (Wang et al., 2018). Interestingly, several lncRNA-based integrated models have been developed for the stratification of AML patients (Liu et al., 2021; Zhu et al., 2023). Recently, an integrated prognostic signature encompassing five immune-related protein-coding genes and an immune-related lncRNA has been successfully constructed to predict the survival and stratification of AML patients (Zhao et al., 2022). Notably, the expression of heat shock proteins (HSPs) is associated with major adverse prognostic factors in AML (Thomas et al., 2005), and some HSP90 inhibitors have been confirmed to be effective agents against primary AML (Flandrin et al., 2008; Lazenby et al., 2015). Likewise, research on ferroptosis-related processes and clinical outcomes in AML is gaining momentum (Zheng et al., 2021; Cui et al., 2022). In addition, some pieces of evidence suggest a link among oncogenes, HSPs, and ferroptosis. For instance, members of the HSP family, such as HSP72/73, HSP70, and HSP90, have been linked to TP53 mutations in numerous cancers (Lane et al., 1993; Sun et al., 1997; Calderwood et al., 2006). Similarly, mutations in RAS and TP53 have been demonstrated as being associated with both HSPs and ferroptosis (Ye et al., 2020; Chen et al., 2021).

Considering that a link between HSPs and ferroptosis in AML has been recently suspected (Dai and Hu, 2022; Liu et al., 2022; Aolymat et al., 2023), herein, we investigated lncRNAs that may act as mediators between two processes like HSPs and ferroptosis in AML. To our knowledge, this is the first computational study integrating these two processes, i.e., HSP and ferroptosis.

## Materials and methods

### Data generation for AML patients from The Cancer Genome Atlas database

Transcriptomic profiling data for AML patients were obtained from The Cancer Genome Atlas (TCGA) database (https://portal.gdc.cancer.gov/repository, TCGA-LAML), while clinical data (cytogenetic risk, age, blast cells, bone marrow blast cells, hemoglobin, leucocytes, FAB classification, and gender) and survival data for AML patients were downloaded from UCSC Xena (https://xena.ucsc.edu/). From the TCGA-LAML dataset, we extracted the gene expression of 97 HSP genes (Supplementary Table S1), 268 ferroptosis genes (Supplementary Table S1), and lncRNAs. Overall, 150 patients were included in our study. By overlapping gene expression data with survival data, 131 patients were included for further analysis. Of these, 84 patients had mutation data and 127 patients had clinical data.

### Identification of HSP and ferroptosis-associated lncRNAs (HSP/ferroptosis-lncRNAs) and construction of a novel prognostic signature

According to Pearson’s correlation analysis, lncRNAs related to HSP genes (HSP-associated lncRNAs) and ferroptosis genes (ferroptosis-associated lncRNAs) were considered on the basis of the following standard: Pearson’s analysis: |R|>0.6 and *p* < 0.001. LncRNAs overlapping between HSPs and ferroptosis-related lncRNAs were designated as HSP-dependent and ferroptosis-related lncRNAs (HSP/ferroptosis-lncRNAs). A total of 131 patients (gene expression and survival data were included) were randomly assigned to a training cohort (n = 66) and a validation cohort (n = 65). A new signature was then determined in the training cohort using the aforementioned HSP/ferroptosis-lncRNAs. In brief, 64 survival-related HSP/ferroptosis-lncRNAs were determined by univariable Cox regression analysis in the training cohort. Cox regression analysis with least absolute shrinkage and selection operator (LASSO) was then used to further test the survival-associated lncRNAs. Based on 10-fold cross-validation and lambda.min values, five lncRNAs were obtained. Multivariate Cox regression analysis based on the minimum value of the Akaike information criterion (AIC) was used to generate a prognostic signature of HSP/ferroptosis-lncRNAs. The signature risk score of each patient was calculated via the following formula: risk score = $\sum_{1}^{n}Coe\;f_{i}\times \mathrm{Exp}\;r_{i}\;\left(Coe\;f_{i}=coefficient,\mathrm{Exp}\;r_{i}=expression\;value\;of\;HSP\;dependent\;ferroptosis\;related\;lncRNA\right)$ . After summarizing the risk scores for the 66 patients, the median risk score was used as a cutoff point to classify them into high- and low-risk groups. It is worth noting that the same cutoff value was also used in the test and overall groups. In addition, a chi-squared test was used to confirm the unbiasedness of the clinical baseline data between the validation (test and overall cohorts) and training cohorts.

### Evaluation of the prognostic signature of the four lncRNAs

The training, test, and overall cohorts were assessed for predictive ability between the high- and low-risk groups using Kaplan–Meier (KM) curves. Receiver operating characteristic (ROC) curves and a concordance index (C-index) were introduced to further validate the predictive ability of the signature in the overall cohort. The CPH function from the ‘rms’ R package was used to perform C-index analysis. Univariable and multivariate Cox regression analyses were used to examine potential independent predictors of survival in the overall cohort by combining signature and clinical characteristics. In addition, this signature was applied to the overall cohort to assess its prognostic potential in subgroups of individual clinical characteristics. Of note, the cutoff values for continuous clinical characteristics were age/60 years, blast cells/median value, bone marrow/median value, hemoglobin/median value, and leucocytes/median value, respectively, while subgroups of the remaining clinical characteristics were cytogenetic risk (favorable/normal vs. poor), FAB classification (non-M3 vs. M3), and sex (male vs. female).

### Functional enrichment analysis

We compared the gene expression of the high-risk and low-risk groups to obtain differential genes that must meet the following criterion: false discovery rate (FDR) < 0.05 and log2-fold change (logFC) > 1. Then, Gene Ontology (GO) enrichment analysis was used to identify biological processes (BPs), cellular components (CCs), and molecular functions (MFs). The Kyoto Encyclopedia of Genes and Genomes (KEGG) analysis was used to explore potential biological signaling pathways.

### Investigating the association of immune function, Tumor Immune Dysfunction and Exclusion, and tumor mutation burden using the obtained signature

Single-sample gene set enrichment analysis (ssGSEA) was used to assess the different immune functions between the high-risk and low-risk groups. The Tumor Immune Dysfunction and Exclusion (TIDE) score can help physicians select patients who are best-suited to receive immune checkpoint therapy, so we calculated the TIDE score of AML patients in TCGA. The TIDE algorithm was used to calculate the TIDE score, and we compared the differential TIDE values between the high- and low-risk groups using the Wilcoxon rank-sum test. We further implemented the R package “maftools” to visualize the mutation profiles of AML patients. The first 16 mutated genes were *TP53, TTN, IDH2, NPM1, DNMT3A, FLT3, ASXL1, KIT, PAN2, FAT2, IDH1, IQCN, KRAS, MUC16, RUNX1,* and *BCORL1*. The difference in the tumor mutation burden (TMB) between the high- and low-risk groups was compared using the Wilcoxon rank-sum test. The differences in survival probability between the high-TMB and low-TMB groups are also presented using KM curves. The optimal cutoff value for the TMB was determined using the surv_cutpoint function in R.

### IC_50_ scores

The determination of the half-maximal inhibitory concentration (IC_50_) serves as a crucial parameter for assessing the effectiveness of and response to a drug treatment. In our study, we utilized the “pRRophetic” package to predict the clinical chemotherapeutic response for each sample.

### Statistical analysis

Statistical analyses were performed using R software. Pearson’s correlation analysis, LASSO Cox regression, univariable and multivariable Cox regression, Kaplan–Meier curves, ROC curves, C-index, and Wilcoxon rank-sum test were used to analyze our study data. *p* < 0.05 was considered significant. *p < 0.05; **p < 0.01; and ***p < 0.001; ns: not significant.

## Results

### Establishing a signature from HSP/ferroptosis-associated lncRNAs (HSP/ferroptosis-lncRNAs) in AML patients

**FIGURE 1:**
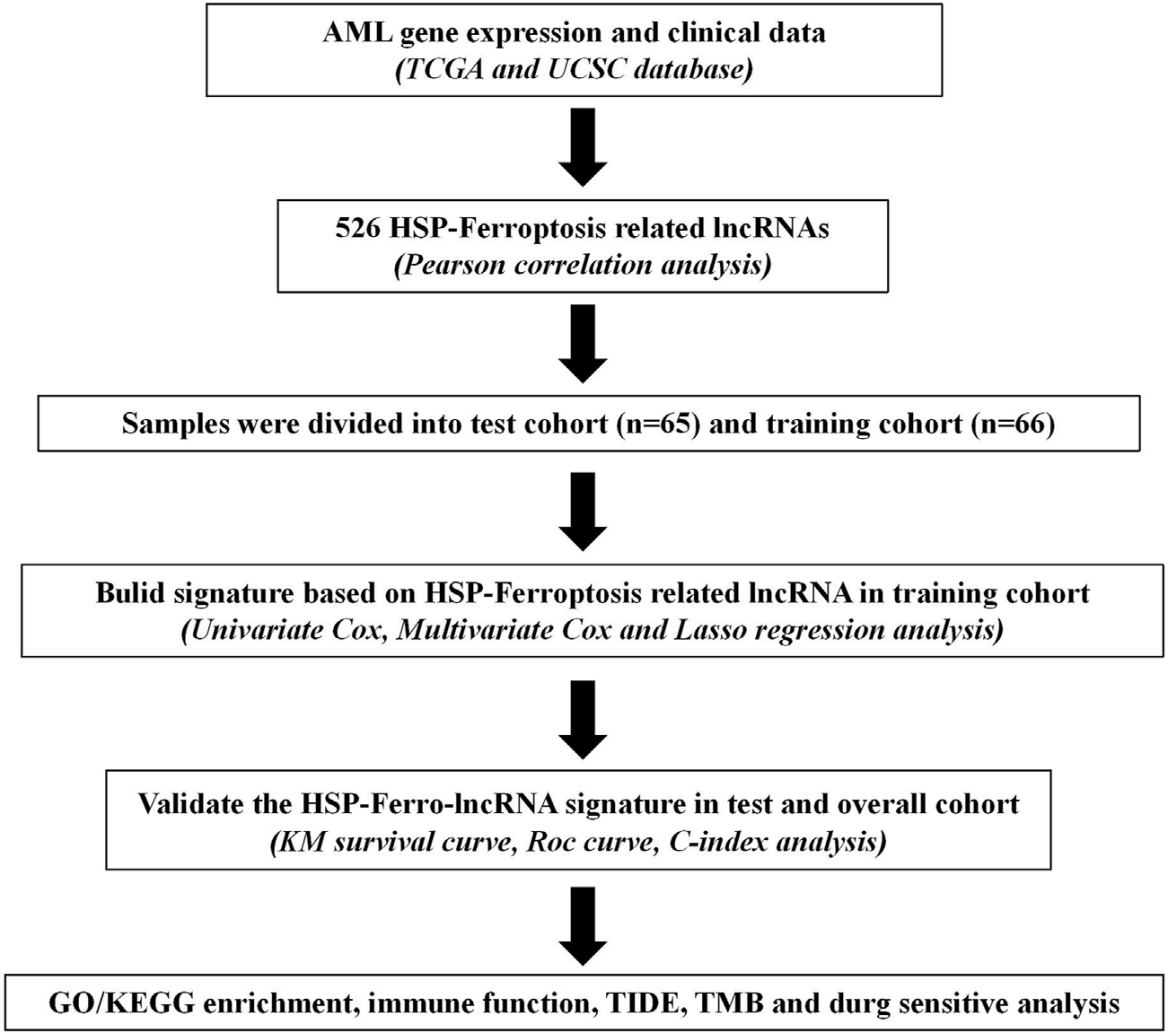
Study flowchart for our analysis. TCGA: The Cancer Genome Atlas; KM curve: Kaplan–Meier curve; ROC: receiver operating characteristic; C-index: concordance index; GO/KEGG: Gene Ontology/Kyoto Encyclopedia of Genes and Genomes; TIDE: Tumor Immune Dysfunction and Exclusion; and TMB: tumor mutation burden.

The flowchart of this study is given in Figure 1. We extracted HSP, ferroptosis, and lncRNA gene expression from the RNA-seq data of AML patients from the TCGA database. Using Pearson’s correlation analysis (|R|>0.6 and *p* < 0.001), 713 lncRNAs associated with HSP genes and 1,537 lncRNAs associated with ferroptosis genes were identified (Supplementary Table S2, 3). Overlapping 526 lncRNAs from the HSP-associated and ferroptosis-associated lncRNAs were determined as HSP/ferroptosis-lncRNAs (Supplementary Table S4). Subsequently, 131 patients (gene expression and survival data) were randomized to the training and test cohorts in a 1:1 ratio. We then determined the prognostic signature in the training group. First, in combination with survival data, we used univariable Cox regression to find the top 64 lncRNAs that were associated with survival time (Supplementary Table S5). To further test the lncRNAs for survival, we used LASSO Cox regression and obtained five lncRNAs (AL138716.1, AC000120.1, AC004947.1, AC020934.2, and LINC01547) (Supplementary Figure S1). In addition, multivariate Cox regression analysis was performed to generate a novel prognostic signature containing four lncRNAs associated with HSP/ferroptosis (AL138716.1, AC000120.1, AC004947.1, and LINC01547) (Table 1). As shown in Supplementary Table S6, all clinical factors were unbiasedly distributed between the training and test cohorts, which was confirmed by using the chi-squared test method (*p*-values >0.05).

**TABLE 1:** Multivariate Cox regression analysis.

LncRNA	Coefficient
AL138716.1	−0.936
AC000120.1	−1.149
AC004947.1	0.658
LINC01547	0.798

### Evaluating and confirming the prognosis of the signature

**FIGURE 2:**
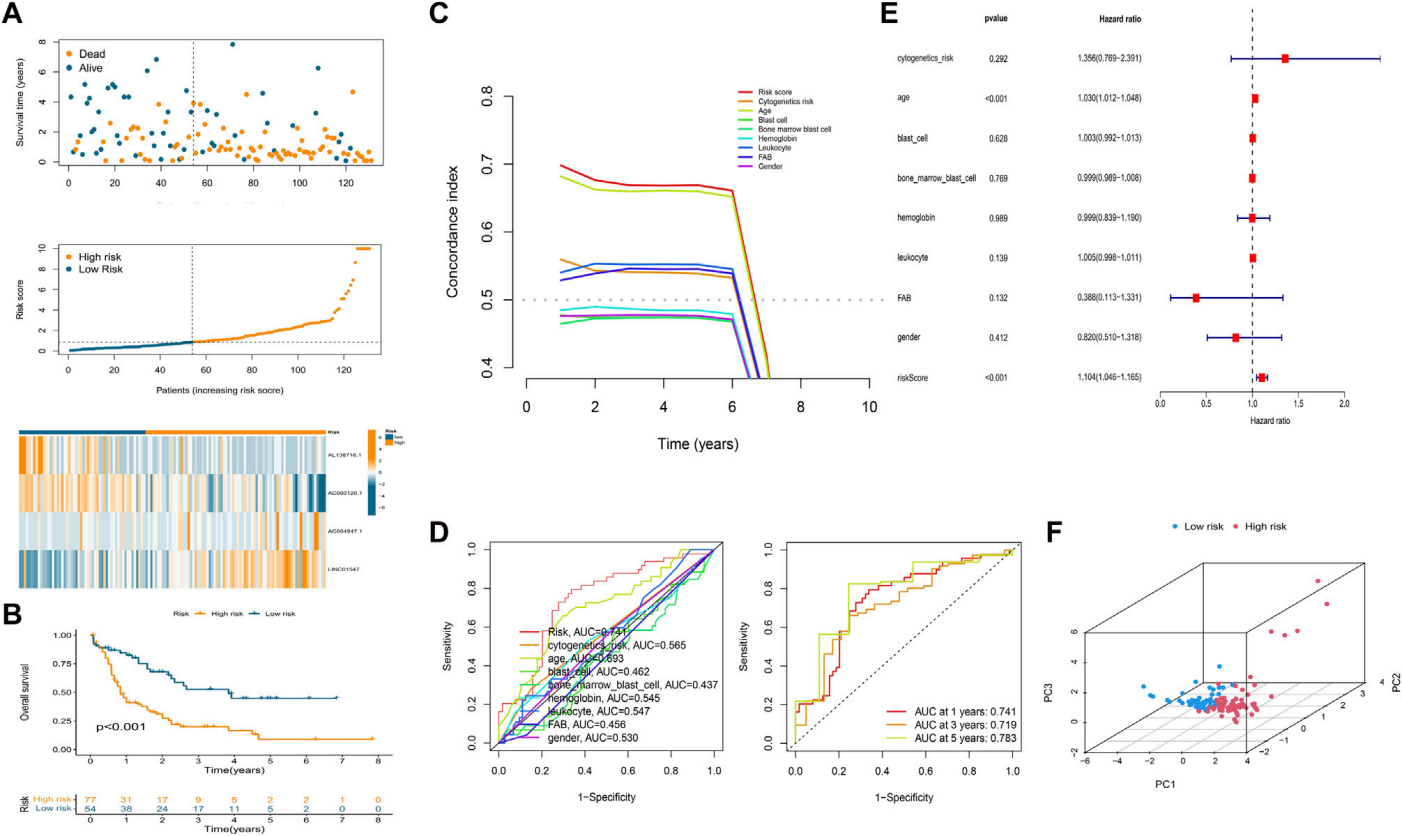
Establishment of a prognostic HSP/ferroptosis-lncRNA signature. **(A)** Survival time, risk score, and heatmap for the overall cohort. **(B)** KM survival curve for AML patients in the overall cohort. **(C)** C-index analysis for the risk score (based on the signature) and clinical characteristics. **(D)** ROC curves of the risk score (based on the signature) and clinical characteristics (left) and ROC curves for risk score (based on the signature) at different time points (1, 3, and 5 years) in the overall cohort. **(E)** Multivariate Cox regression analysis. **(F)** PCA of the HSP/ferroptosis-lncRNA signature.

We calculated the risk score of each patient using the formula given in Materials and Methods. According to the median value of the risk score of the patients in the training cohort, we classified the patients in the three cohorts (training, test, and overall cohorts) into high- and low-risk groups. The risk level, survival status, and survival time between the high- and low-risk groups in these three cohorts are shown in Figure 2 and Supplementary Figure S2. The expression of the four lncRNAs associated with HSP and ferroptosis for each patient in the different cohorts is shown as a heatmap (Figure 2A and Supplementary Figure S2A). Survival analysis (KM method) showed that the OS of the low-risk group was longer than that of the high-risk group in the training cohort (*p* < 0.001), test cohort (*p* = 0.046), and overall cohort (*p* < 0.001) (Figure 2B and Supplementary Figure S2B). The prognostic significance of the signature was further confirmed using the ROC curve and C-index analysis in the overall cohort. As shown in Figure 2C, the risk score based on our signature and age showed values greater than 0.65 for the C-index method, and the risk score is higher than the age. Moreover, compared with other clinical characteristics, the risk score has the highest AUC (0.741) in the ROC curve (Figure 2D). In addition, the AUC values of the ROC curve at different time points were all above 0.700 at 1 year (AUC = 0.741), 3 years (AUC = 0.719), and 5 years (AUC = 0.783) (Figure 2D). Thus, the signature represents a robust model for predicting survival in AML patients.

Univariable cox regression was used to confirm that the risk score (based on the signature), FAB classification, and age are factors that can predict survival in AML patients (Supplementary Figure S2C). In addition, by combining the risk score (signature-based) and clinical characteristics, we confirmed the risk score and age to be independent predictors of survival in AML patients using multivariate Cox regression (Figure 2E). PCA was then performed to test the ability to cluster the high- and low-risk patients in different groups including the HSP/ferroptosis-lncRNA signature, overall gene expression profile, HSP genes, HSP-associated lncRNAs, ferroptosis genes, ferroptosis-associated lncRNAs, and HSP-associated and ferroptosis-associated lncRNAs. Supplementary Figure S3 and Figure 2F show that the HSP/ferroptosis-lncRNA signature group showed a significant distribution between the high- and low-risk subgroups, whereas the other groups were relatively dispersed between the high- and low-risk subgroups. These results showed that the prognostic signature can discriminate well between high- and low-risk groups.

**FIGURE 3:**
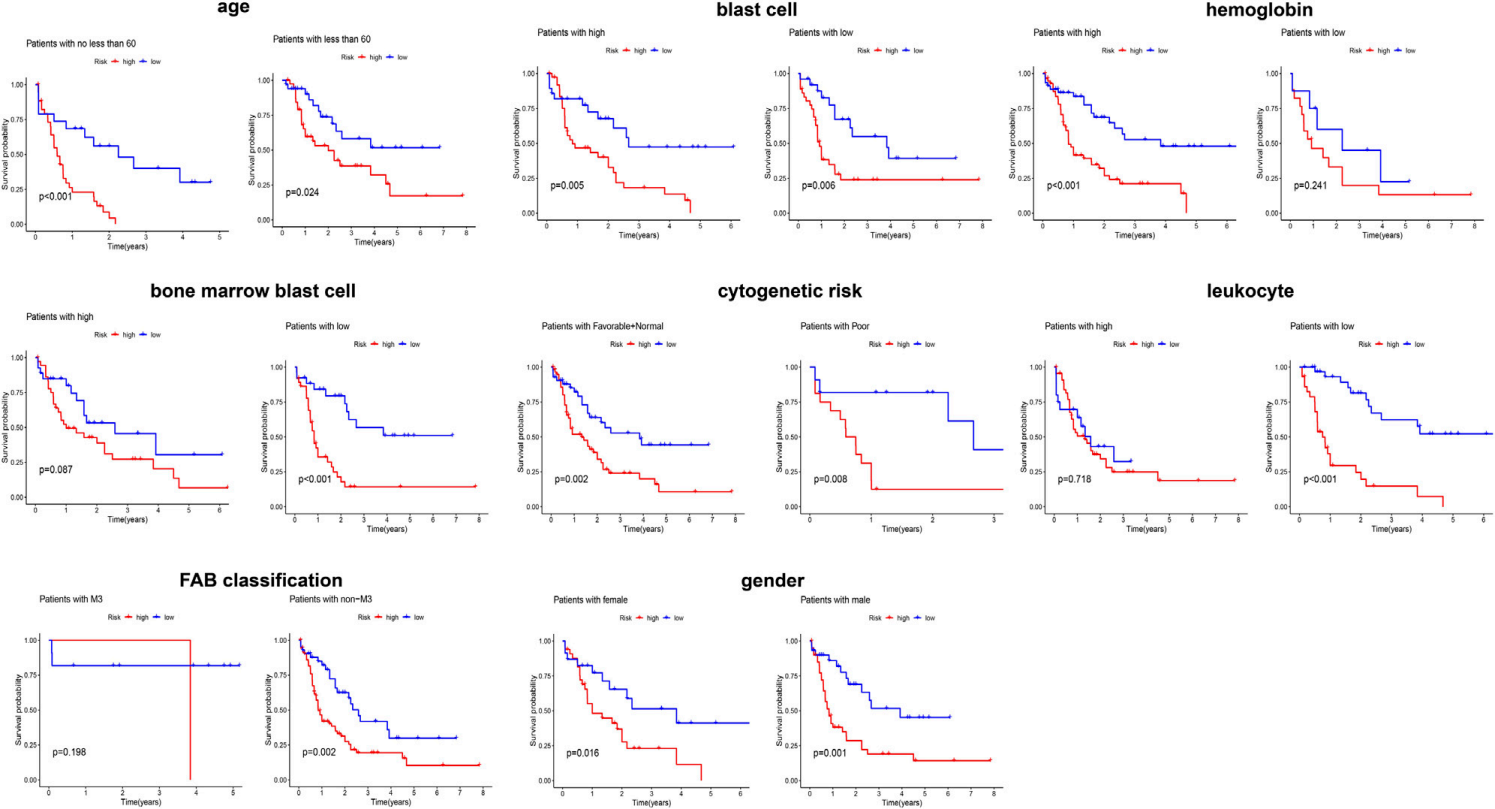
Evaluating the predicting ability of the obtained signature in clinical subgroups using KM curves.

Given that elderly patients, patients with high leukocyte value and/or poor cytogenetics risk, etc., have poor prognosis in clinic, we further assessed the predictive ability of the obtained signature in clinical subgroups using KM curves (Figure 3). We divided the clinical characteristics into the following subgroups: sex (male and female), age (≥60 and <60), FAB (M3 and non-M3) and cytogenetic risk (favorable + normal and poor), blast cells (high and low), bone marrow blast cells (high and low), hemoglobin (high and low), and leucocytes (high and low). Of note, the classification of blast cells, bone marrow blast cells, hemoglobin, and leucocytes into high and low groups was based on their median value. After applying the obtained signature to classify the patients into low and high risk, the differential survival probability between low- and high-risk patients was shown as gender (male, *p* = 0.001), gender (female, *p* = 0.016), age (≥60, *p* < 0.001), age (<60, *p* = 0.024), FAB (M3 group, *p* = 0.198), FAB (non-M3, *p* = 0.002), cytogenetic risk (favorable + normal, *p* = 0.002), cytogenetic risk (poor, *p* = 0.008), blast cells (high, *p* = 0.005), blast cells (low, *p* = 0.006), bone marrow blast cells (high, *p* = 0.087), bone marrow blast cells (low, *p* < 0.001), hemoglobin (high, *p* < 0.001), hemoglobin (low, *p* = 0.241), leucocytes (high, *p* = 0.781), and leucocytes (low, *p* < 0.001) in Figure 3. Overall, the signature has prognostic ability in most clinical subgroups.

### Correlation of functional enrichment, immune function, mutations, and TIDE analysis with the obtained signature

**FIGURE 4:**
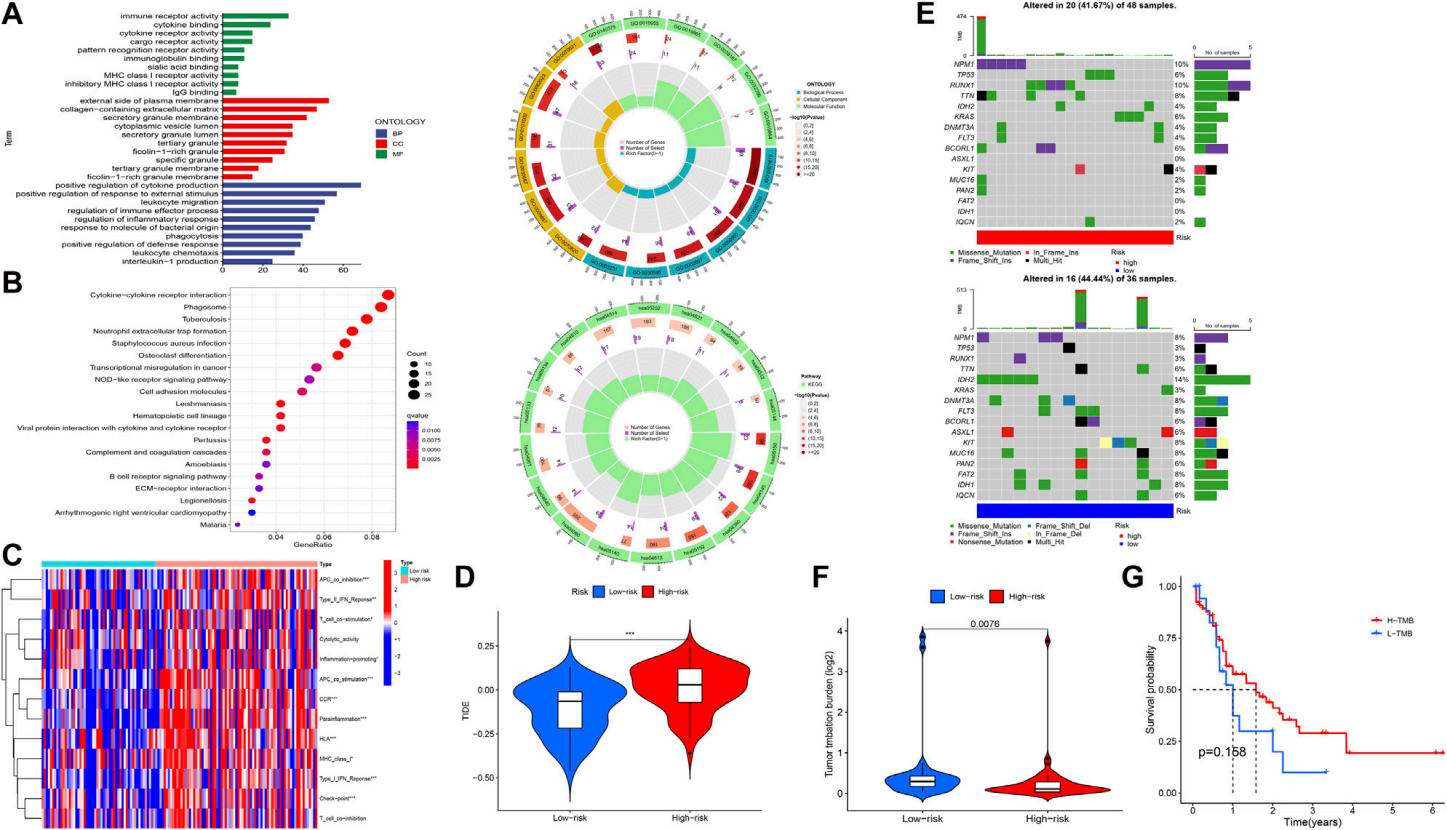
Functional enrichment and immune and mutation-associated analysis. **(A, B)** GO and KEGG enrichment analysis. **(C, D)** Differential immune indicators and TIDE scores between low-risk and high-risk groups based on the obtained signature. **(E)** Waterfall plot showing the mutation landscape of high-risk and low-risk group AML patients. **(F)** Difference in the TMB between high-risk and low-risk groups. **(G)** Survival probability between high- and low-expression TMB.

To elucidate the relationship between the signature and potential function (BP, CC, MF, and pathway), we performed functional enrichment analysis using the GO and KEGG method (Figures 4A,B). Interestingly, the enrichment analysis revealed the involvement of many immune-related BPs, MFs, and pathways for the signature. To examine changes in immune markers between the high- and low-risk groups based on our signature, we used the ssGSEA and Wilcoxon rank-sum test (Figure 4C). The results showed that APC inhibition/stimulation, interferon (IFN) type I/II responses, chemokine receptor (CCR), para-inflammation, human leukocyte antigen (HLA), major histocompatibility complex (MHC) class I, checkpoint, T-cell stimulation, and promotion of inflammation were significantly more active in the high-risk group than in the low-risk group. Thus, this signature is implicated in the immune progression/functioning in AML patients. Considering this, we further investigated the association of the immune checkpoint blockade with the signature using TIDE analysis (Figure 4D). The high-risk group showed a high TIDE score compared to the low-risk group, suggesting a lower sensitivity to immune checkpoint inhibitors in the high-risk group, which could help in predicting ICI treatment in the clinic for patients classified based on the signature.

Given that gene mutations are an important part of AML, we analyzed the mutation data in our study. The 16 most mutated genes from 84 samples (samples that contained gene mutation data) were used in a high-risk group (48 samples) and a low-risk group (36 samples) to assess the differential mutation landscapes, as shown in Figure 4E. In particular, the mutation rates of the NPM1 and RUNX1 genes in the high-risk group and the IDH2 gene in the low-risk group were 10%, 10%, and 14%, respectively. As shown in Figure 4F, the TMB estimates in the low-risk group exceeded those in the high-risk group (*p* = 0.0076). However, there was no difference in survival time between the high- and low-risk groups with respect to the TMB (Figure 4G).

### IC_50_ scores

**FIGURE 5:**
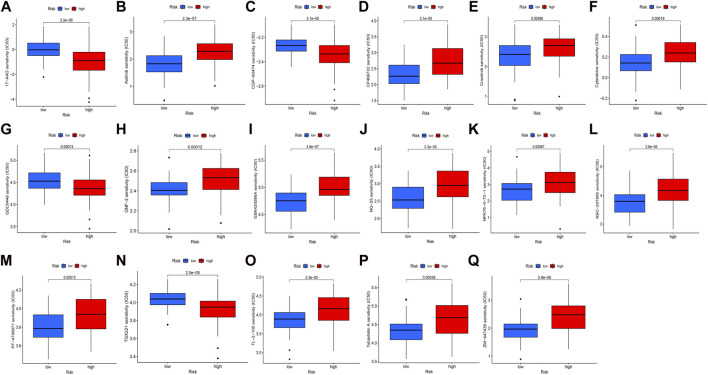
Distribution of IC_50_ scores of targeted drugs in different HSP-ferroptosis-related lncRNA risk groups. **(A)** 17-AAG, **(B)** axitinib, **(C)** CGP-60474, **(D)** CP466722, **(E)** crizotinib, **(F)** cytarabine, **(G)** GDC0449, **(H)** GNF-2, **(I)** GSK429286A, **(J)** NG-25, **(K)** NPK76-II-72–1, **(L)** NSC-207895, **(M)** PF-4708671, **(N)** TGX221, **(O)** TL-2-105, **(P)** tubastatin A, and **(Q)** ZM-447439.

In our study, we examined the differences in the IC_50_ scores for chemotherapy between high- and low-risk groups based on the obtained signature. Specifically, we observed that the IC_50_ values for axitinib, CP466722, crizotinib, cytarabine, GNF-2, GSK429286A, NK-25, NPK76-II-72–1, NSC-207895, PF-4708671, TL-2-105, tubastatin, and ZM-447439 were higher in the high-risk group. Conversely, the IC_50_ values for 17-AAG, CGP-60474, GDC0449, and TGX221 were lower in the high-risk group (Figure 5). These findings support the notion that there is a statistically significant difference in the distribution of IC_50_ values for targeted agents among high- and low-risk groups based on the obtained signature.

## Discussion

Although studies in recent decades have improved our understanding of AML, the underlying pathogenesis of this lethal disease has not yet been fully elucidated. With the development of NGS technologies, more and more AML-related mechanisms have emerged, including the eventual contribution of long noncoding RNAs (lncRNAs) (Mer et al., 2018; Liu et al., 2019; Mishra et al., 2022). In fact, several studies have established lncRNA-based prognostic models for clinical characterization in AML patients (Zhao et al., 2021; Ding et al., 2022; Li et al., 2022; Zhang et al., 2022). Independently, an association of HSPs (Li and Ge, 2021) and ferroptosis-related lncRNAs (Zheng et al., 2021) has been demonstrated in AML. Given that HSPs and ferroptosis appear to be closely linked to tumorigenesis (Liu et al., 2022), using a comprehensive bioinformatics approach, we sought to identify lncRNAs that may overlap with these processes with predictive relevance for AML patients.

To determine this, we extracted the HSP-, ferroptosis-, and lncRNA-related gene expression data of AML patients using the TCGA database. Using Pearson’s correlation analysis, we identified overlapping lncRNAs (termed HSP/ferroptosis-lncRNAs), and subsequent analysis revealed four lncRNAs associated with HSP/ferroptosis genes (AL138716.1, AC000120.1, AC004947.1, and LINC01547) as a prognostic signature. In particular, AC000120.1 has been recently reported in a prediction model based on seven cuproptosis-related lncRNAs for AML prognosis (Zhu et al., 2023). Similarly, LINC01547 has been reported in m6A-related lncRNAs associated with prognosis and immune response in AML patients (Li et al., 2021). While AC004947.1 has shown oncogenic potential (Zhao et al., 2020), AL138716.1 has not yet been reported in studies. Notably, when we tested the obtained signature to classify AML patients, we found that high-risk patients had a lower survival probability compared to the low-risk group, indicating the prognostic ability of the signature in AML, and the following analysis confirms that the signature is a robust independent factor for AML patients. In addition, the prognostic ability also presents its potential predicting ability in different clinical subgroups.

Both GO and KEGG analysis provided immune-related evidence in AML. Some immune indicators in possible different between low-risk and high-risk group. Moreover, the high-risk group showed a high TIDE score, indicating a lower sensitivity to immune checkpoint inhibitors in the high-risk group, potentially helping to predict ICI treatment in the clinic for patients classified on the basis of the signature. Overall, these lines of evidence revealed the relation of the obtained signature with immune response. The differential landscapes of gene mutation and tumor mutational burden were found between high- and low-risk groups, which may partly contribute to the prognostic ability of our signature. Furthermore, our study contributes valuable insights into the varying treatment sensitivity among AML patients by conducting drug sensitivity analysis for the high-risk and low-risk groups based on the HSP–ferroptosis–lncRNA status.

It is also worth noting the limitation to this study, as the analysis relies purely on comprehensive bioinformatics and requires effective experimental validation. Nevertheless, two out of four lncRNAs in our signature have been proven in AML, thus providing evidence that our predictive model of lncRNA may correlate with the processes of HSPs and ferroptosis in AML.

## Data Availability

The original contributions presented in the study are included in the article/Supplementary Material; further inquiries can be directed to the corresponding author. Publicly datasets utilized in this study can be accessed via The Cancer Genome Atlas (https://portal.gdc.cancer.gov/, Project: TCGA-LAML) and Ucsc Xena (https://xenabrowser.net/datapages/, Cohort: GDC TCGA Acute Myeloid Leukemia (LAML)).
